# Word correlation matrices for protein sequence analysis and remote homology detection

**DOI:** 10.1186/1471-2105-9-259

**Published:** 2008-06-03

**Authors:** Thomas Lingner, Peter Meinicke

**Affiliations:** 1 Department of Bioinformatics, Institute of Microbiology and Genetics, Georg-August-University Göttingen, Göttingen, Germany

## Abstract

**Background:**

Classification of protein sequences is a central problem in computational biology. Currently, among computational methods discriminative kernel-based approaches provide the most accurate results. However, kernel-based methods often lack an interpretable model for analysis of discriminative sequence features, and predictions on new sequences usually are computationally expensive.

**Results:**

In this work we present a novel kernel for protein sequences based on average word similarity between two sequences. We show that this kernel gives rise to a feature space that allows analysis of discriminative features and fast classification of new sequences. We demonstrate the performance of our approach on a widely-used benchmark setup for protein remote homology detection.

**Conclusion:**

Our word correlation approach provides highly competitive performance as compared with state-of-the-art methods for protein remote homology detection. The learned model is interpretable in terms of biologically meaningful features. In particular, analysis of discriminative words allows the identification of characteristic regions in biological sequences. Because of its high computational efficiency, our method can be applied to ranking of potential homologs in large databases.

## Background

Advances in large-scale sequencing have led to a vast amount of protein sequences that have to be classified into structural and functional classes. Because experimental determination is time consuming and expensive, several computational methods based on sequence similarity were introduced to automatically annotate sequences by homology transfer. For close homologs, i.e. sequences with a similarity of more than 80% at the amino acid level, this can be done by pairwise comparison methods like the Smith-Waterman local alignment algorithm [1] or BLAST [2]. However, these methods often fail in cases where sequence similarity is low. In the so-called "twilight-zone", the detection of remote homologies still remains a challenging task in computational biology.

Remote homology detection methods are often based on a statistical representation of protein families and can be divided into two major categories: first, profile-based methods provide a non-discriminative approach to family-specific representation of sequence properties. The corresponding generative models are usually trained using only known example sequences of the particular family [3,4]. Second, discriminative methods provide a supervised approach [5-8] to representing sequence properties that explicitly model the differences between protein families. In this case, training requires example sequences from the particular protein family and counterexamples from the other protein families.

Discriminative methods often measure the similarity of two sequences by means of a kernel function. A sequence kernel computes the inner product of sequence representatives in some abstract feature space, often without explicit transformation of the sequences into that space. Using learning algorithms that only need to evaluate inner products between feature space elements, the "kernel trick" makes learning in complex and high dimensional feature spaces possible. Recent studies [7-14] have shown that discriminative kernel methods can significantly increase the detection performance as compared with profile-based methods.

Kernel methods in general require the evaluation of *N*^2 ^kernel functions for training the discriminant function on a set of *N *sequences. Since this requirement is computationally demanding even for a few thousand sequences, the use of kernel-based approaches for large-scale discriminative learning is problematic. Testing the trained model is also expensive since it involves kernel computations between test examples and *N *training examples.

However, in some cases evaluation of the discriminant can be computed rather efficiently if an explicit representation of the discriminant in feature space is possible. For example, the Spectrum kernel [9] measures the similarity between two sequences by counting the occurrences of all *K*-length subsequences ("*K*-mers") in these sequences. The method has been shown to provide considerable speed-up of the evaluation using the discriminant in the *K*-mer feature space. However, the use of the Spectrum kernel for longer *K*-mers is problematic, because of the decreasing number of perfect matches. Several methods based on inexact matches have been introduced to tackle this problem [15]. These methods count the occurrences of nearly matching *K*-mers by means of a binary match function that is invariant with respect to changes within a specified "mutation neighborhood". For example, the Mismatch kernel [8] defines a mapping to the *K*-mer feature space via a (*K*, *m*)-"mismatch neighborhood", i.e. the occurrence of a particular *K*-mer *i *contributes to all feature space dimensions associated with *K*-mers that differ from *i *by at most *m *mismatches. Recently, Oligomer Distance Histograms [14] have been introduced for protein sequence representation and remote homology detection. Here, the similarity between two sequences is measured by counting the occurrences of all *K*-mer pairs for all distances. Oligomer Distance Histograms are highly competitive with state-of-the-art methods for remote homology detection and provide an explicit feature space. All these feature-based methods allow for fast classification of new sequences. Furthermore, they do not require prior knowledge about sequence properties in terms of relevant motifs or structural information. By analysis of the discriminative features, these methods can even help to find new motifs or other interesting sequence properties.

In contrast, motif kernels [7] evaluate the occurrences of known motifs from an existing motif database, i.e. the number of matching motifs in a pair of sequences is used to define a kernel. As another example, profile kernels [11] use probabilistic profiles as produced by PSI-BLAST to define "positional mutation neighborhoods", i.e. profile-defined mappings to the *K*-mer feature space. Here, the profiles originate from an initial homology search of training examples, therefore this method can also be viewed as a homology-based kernel. Based on prior knowledge, motif kernels and profile kernels also provide an explicit representation of the discriminant, and thus allow for interpretation in the associated feature space and fast classification of new sequences.

Currently, alignment-based kernels show the best detection performance on widely-used homology detection setups [10,12]. For example, in [10] the authors derive the similarity measure between two sequences from the sum of their local alignment scores. This similarity measure requires additional transformation in order to provide a valid kernel. However, these methods show a significant disadvantage concerning the *interpretability *of the resulting discriminant model. In contrast to methods that are based on a meaningful vector space representation of the sequences, alignment-based kernels do not provide direct inspection of the associated feature space. With this limitation it is difficult to identify the relevant sequence properties that have been learned from the data. Therefore, these kernels do not offer additional utility for researchers interested in finding the characteristic features of protein families. In principle, the same holds for kernel methods that involve certain kinds of nonlinear transformations, like Gaussian (RBF) kernels do, because the learned discriminant parameters, i.e. the sequence-specific weights after kernel-based training, cannot be associated with particular sequence properties. This considerably complicates the interpretation of these "black box" classification models.

As an additional drawback, several kernel methods incorporate *hyperparameters *that have to be carefully adjusted before training. For example, the authors of [10] used a total number of 3 kernel parameters, two of which were fixed in an ad-hoc manner. The dependence of the performance on the third parameter was evaluated on the test data in this particular setup. Other approaches, e.g. [12] and [13] also comprise several hyperparameters that were chosen to provide maximum performance on the test data. The extensive use of hyperparameters increases the risk of overfitting when no dedicated validation data set is used. In this case, the application of the method to different data is difficult because new data are likely to require the readjustment of these parameters.

In this work, we present an alignment-free feature space representation for protein sequences, which is based on the average pairwise similarity of short subsequences ("words"). First, we show that this similarity measure defines a valid kernel function between two sequences. We then provide some further analysis of the associated sequence representation, which gives rise to a well interpretable feature space in terms of "word correlation matrices" (WCMs). Finally, we demonstrate the performance of this representation on a widely-used benchmark setup for protein remote homology detection. In addition, we show how the resulting discriminants can be analyzed to gain insight into particular sequence properties.

## Methods

### From Average Word Similarity to Word Correlation Matrices

We first define a sequence similarity measure based on average word similarity. Consider two sequences *S*, $\tilde{S}$, represented by two lists of words *W*, $\tilde{W}$ containing all consecutive overlapping *K*-length words *w*_*i*_, ${\tilde{w}}_{j}$ occurring in the respective sequence(s). With some word similarity function *s*(*w*, $\tilde{w}$) measuring the similarity between words *w *and $\tilde{w}$ we compute the *average word similarity *between sequences *S*, $\tilde{S}$ by

(1) $$k(S,\tilde{S})=\frac{1}{n\tilde{n}}\sum_{i=1}^{n}\sum_{j=1}^{\tilde{n}}s(w_{i},{\tilde{w}}_{j})$$

where *n *and *ñ *denote the number of *K*-length words in the sequences. In particular we are interested in word similarity functions that provide a positive semidefinite sequence similarity measure, i.e. that provide valid sequence *kernels*. We here propose a simple realization of the word similarity function that not only results in a valid sequence kernel but also implies a feature space of moderate dimensionality. Consider an alphabet A and a binary vector encoding of *K*-length words **x **∈ ${\left\{0,1\right\}}^{K|A|}$. The *i *- *th *letter of a word only yields a non-zero entry in vector dimension *K *× (*i *- 1) + *j *if that letter matches the *j*-th element of the alphabet. Let **z **∈ {0, 1}^20 ^be an amino acid indicator vector, i.e. a 20-dimensional vector that contains only one non-zero entry for the vector dimension associated with a particular amino acid. With this definition and ^*T *^indicating vector (matrix) transposition, a word vector for protein sequences corresponds to a stacking of particular amino acid indicator vectors $x={\left[z_{1}^{T},...,z_{K}^{T}\right]}^{T}$ for *K *different word positions. With the two word vectors **x**, $\tilde{x}$ of the words *w*, $\tilde{w}$ our word similarity is computed by the squared dot product

(2) $$s(w,\tilde{w})={\left(x^{T}\tilde{x}\right)}^{2}.$$

Note that this measure corresponds to the squared number of matching letters occurring at the same position in both words. In terms of the Hamming distance *h*(*w*, $\tilde{w}$) between words, it is equal to (*K *- *h*(*w*, $\tilde{w}$))^2^. We shall now show that this formulation gives rise to a valid sequence kernel *k*(*S*, $\tilde{S}$) if used in Equation (1). Further we will consider the dimensionality of the associated feature space, which will be shown to grow quadratically with the word length *K*. We now write the above sequence similarity in terms of the word vectors **x**_*i *_and ${\tilde{x}}_{j}$ of *S *and $\tilde{S}$, respectively:

(3) $$k(S,\tilde{S})=\frac{1}{n\tilde{n}}\sum_{i=1}^{n}\sum_{j=1}^{n}{\left(x_{i}^{T}{\tilde{x}}_{j}\right)}^{2}$$

(4) $$=\frac{1}{n\tilde{n}}\sum_{i=1}^{n}\sum_{j=1}^{\tilde{n}}\left(x_{i}^{T}{\tilde{x}}_{j})({\tilde{x}}_{j}^{T}x_{i}\right)$$

(5) $$=\frac{1}{n\tilde{n}}\sum_{i=1}^{n}\sum_{j=1}^{\tilde{n}}\text{tr}(x_{i}x_{i}^{T}{\tilde{x}}_{j}{\tilde{x}}_{j}^{T})$$

(6) $$=\text{tr}\left(\underset{S-specific}{\underset{︸}{\frac{1}{n}\sum_{i=1}^{n}x_{i}x_{i}^{T}}}\underset{\tilde{S}-specific}{\underset{︸}{\frac{1}{\tilde{n}}\sum_{j=1}^{\tilde{n}}{\tilde{x}}_{j}{\tilde{x}}_{j}^{T}}}\right)$$

where *tr *denotes the trace function, i.e. the sum of diagonal elements. With matrix **X**_*S *_containing all word vectors **x**_*i *_of sequence *S *as columns, we define the sequence-specific *word correlation matrix *(WCM) as

(7) $$C(X_{S})=\frac{1}{n}\sum_{i=1}^{n}x_{i}x_{i}^{T}=\frac{1}{n}X_{S}X_{S}^{T}$$

With the abbreviations **C **≡ **C**(**X**_*S*_) and $\tilde{C}\equiv C(X_{\tilde{S}})$ we can finally write the kernel as

(8) $$k(S,\tilde{S})=\text{tr}(C\tilde{C})=\text{vec}{\left(C\right)}^{T}\text{vec}(\tilde{C}).$$

The *vec *function converts a matrix to a vector by stacking the matrix columns successively, i.e. the upper right element in a 2 × 2 matrix contributes to the third vector dimension. From this we see that the sequence kernel corresponds to a dot product in a particular feature space which arises from vectorized WCMs. In the following, we use

(9) **Φ **= vec(**C**)

to denote the feature space representative of a sequence.

### WCM feature space

The particular primary structure of a protein is commonly characterized by a sequence of amino acids. The IUPAC one-letter abbreviation code for 20 naturally occurring amino acids gives rise to an alphabet A = {*A*, *R*, *N*, ..., *V*} with |A| = 20. For a protein sequence *S *and a given word length *K*, every dimension in the WCM feature vector **Φ **corresponds to the number of occurrences of two particular amino acids at specific positions within all words of length *K *in *S*. For example, the first feature space dimension counts the occurrences of Alanine (*A*) at the first position of all words. The second dimension corresponds to the number of occurrences of Alanine *and *Arginine at the first position. If the binary **z**-vector encoding as defined in the previous section is used, this dimension always contains a zero value, because different amino acids cannot occur at the same word position by definition. However, this dimension can be useful in combination with word encoding schemes that take into account amino acid substitutions. As a last example, the 21st dimension in our WCM feature space corresponds to the number of occurrences of Alanine at the first and second position of all words, i.e. the frequency of the dimer *AA*.

Interestingly, the features of the WCM representation correspond to features of special realizations of Oligomer Distance Histograms [14]: for a particular word length *K *the WCM features correspond to features of Monomer Distance Histograms when only distances up to *K *- 1 are taken into account. For a particular distance *D*, Monomer Distance Histograms contain the number of occurrences of all amino acid pairs whose sequence positions differ by *D*. A feature in the WCM feature space contains the number of occurrences of two amino acids at distance *D *at particular positions within the same word. Because of overlapping words in a sequence, a particular feature associated with a dimension in the Monomer Distance Histogram feature space is counted at most *K *times and added to different WCM feature space dimensions according to specific word positions. On the other hand, the first and last *K *- 1 words in a sequence have less overlap with other words than words inside the sequence, such that features of words at the beginning and at the end of a sequence are counted less than *K *times. Therefore, long words and short sequences would result in more different features as compared with the Monomer Distance Histogram feature space. In total, the WCM feature space comprises (*K*|A|)^2 ^dimensions, and thus grows quadratically with the word length. Because of the symmetry of the WCM, it is sufficient to consider the upper (or lower) triangular matrix, which can be used to reduce the dimensionality of the feature space to $\frac{K|A|(K|A|+1)}{2}$. Furthermore, off-diagonal elements of entries belonging to the same word position can be disregarded if amino acid indicator vectors are used. In this case, the feature space reduces to $K|A|+\frac{K|A|(K|A|-1)}{2}$ dimensions.

### Kernel matrix computation

For kernel-based training with a set of *N *sequences, the *N *× *N *matrix of pairwise kernel functions between all sequences has to be computed. Doing this directly according to Equation (3) requires $\frac{N(N-1)}{2}$ evaluations of all *L*$\tilde{L}$ word similarity values between two sequences of length *L *and $\tilde{L}$, respectively. Therefore, the overall algorithmic time complexity of this method is *O*(*N*(*N *- 1)*L*$\tilde{L}$*K*|A|). With *L *≈ $\tilde{L}$ and |A| = *const*. this simplifies to *O*(*N*^2^*L*^2^*K*). In particular, for long sequences this can be computationally demanding.

However, in most cases the kernel matrix can be efficiently calculated using the feature space representatives **Φ **of the sequences as defined in Equation (9). After transformation of all sequences into the WCM feature space, their representatives can be stored in a matrix **M **= [**Φ**_1_, ..., **Φ**_*N*_]. Then, the kernel matrix **K **can be computed by the matrix product

(10) **K **= **M**^*T*^**M**.

Using the same simplifications as above, the feature-based computation of the kernel matrix involves *N *sequence transformations of complexity *O*(*LK*^2^) and the evaluation of the matrix product involving the *LK*^2 ^× *N *matrix **M**, which is of theoretical complexity *O*(*N*^2^*LK*^2^). Therefore, the overall time complexity of this method is *O*(*N*^2^*LK*^2^). In contrast to the direct kernel computation, the computational complexity only grows linearly with the length of the sequences but quadratically with the word length.

The theoretical overall time complexity formulas indicate that for *L *> *K *the feature-based method is preferable for calculation of the kernel matrix. In general, *K *has to be chosen to be significantly smaller than *L *in order to obtain reasonable sequence similarity values. Feature-based calculation is much more efficient than the direct computation for moderate word length *K*. However, the memory requirements to store all feature vectors grows quadratically with the word length *K*.

We compared the required time for computation of the kernel matrix using 1000 protein sequences with an average length of 118.6 amino acids. The feature-based calculation using a word length of *K *= 5 (*K *= 10) took 3.09 (7.51) seconds on an AMD Opteron 870 processor with 2GB RAM. Thereby 1.83 (3.62) seconds were used for the transformation of the sequences into the 5050 (20100) dimensional feature space and 1.26 (3.89) seconds were used for the computation of the matrix product. In contrast, the direct calculation of the kernel matrices took 583 and 927 seconds, respectively.

### Discriminant function in feature space

After kernel-based training, the learned sequence-specific weights can be used to calculate the discriminant weight vector in WCM feature space for better interpretation and fast computation of the discriminant.

Let ***α ***= [*α*_1_, ..., *α*_*N*_]^*T *^be the weight vector of a set of *N *sequences after kernel-based training and **M **be the matrix of sequence representatives. Then, the discriminant weight vector **w **in feature space can be computed according to

(11) **w **= **M*α***.

The magnitude of an entry in **w **reflects the discriminative power of the corresponding feature. This can be used to identify relevant features or feature combinations for a given set of sequences. For better interpretability, the discriminant weight vector can be remapped to the WCM space, which provides a convenient visualization of the discriminant.

The discriminant weight vector in feature space can also be used to identify discriminative words in a set of sequences. The discriminative power of a particular word in terms of a word score *score*(**x**) can be computed with the discriminant weight vector **w **and the word vector **x **according to

**Figure 1:**
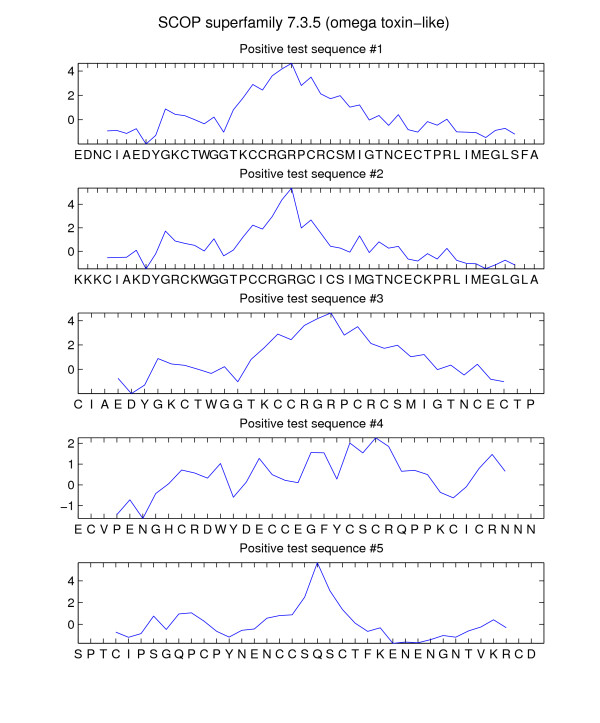
**Word score profiles for positive test sequences of SCOP superfamily 7.3.5**. Word score profiles of the first 5 positive test sequences associated with experiment 1 (SCOP superfamily 7.3.5: omega toxin-like) using word length *K *= 6. Amino acid sequences are mapped to the x-axis while the y-axis corresponds to discriminative word scores. Word score values are centered at position 4 of the overlapping words. See Equation (12) in section "Discriminant function in feature space" for details about calculation of word scores.

(12) *score*(**x**) = **x**^*T*^**Wx **

where **W **is the WCM space representation associated with **w**, i.e. vec(**W**) = **w**. High absolute word score values indicate importance of *w *for discrimination between positive and negative example sequences. These discriminative words can be interpreted biologically in terms of short "motifs", i.e. conserved sequence regions within a set of related sequences. Scores with a low magnitude usually correspond to words that do not contribute significantly to the discrimination, e.g. words that occur in positive and in negative example sequences. Discriminative word scores can also be used to detect discriminative regions within sequences by means of score profiles. A score profile of a sequence *S *is the sequence of word scores for all overlapping words of *S*. Discriminative regions of *S *correspond to global or local maxima (minima) of the score profile of *S*. In Figure 1, five exemplary word score profiles are shown.

For fast classification of a new sequence *S *with the discriminant weight vector in WCM feature space, the classification score can be efficiently computed according to

(13) *Score*(*S*) = **w**^*T*^**Φ **.

The score computation involves transformation of the sequence to the feature space with complexity *O*(*LK*^2^) and the calculation of the dot product for at most (*K*|A|)^2 ^vector elements. Using the same simplification as in the previous section, the overall computational complexity of classification with the feature space discriminant is *O*(*LK*^2^). In contrast, for kernel-based classification of *S *the evaluation of *N *kernel functions

(14) $$Score(S)=\sum_{i=1}^{N}\alpha_{i}k(S_{i},S)$$

according to *N *training sequences is necessary. Note that only kernels with a non-zero *α*_*i *_(support vectors) need to be considered. With *L*^2^*K *computations for evaluation of a single kernel function the overall complexity for kernel-based classification is *O*(*NL*^2^*K*). This indicates that for large *N *the feature-based computation of the classification score can be faster by orders of magnitude.

## Results

In order to evaluate our approach, we considered a widely-used benchmark data set for remote homology detection [6] based on the SCOP database [16]. In the corresponding setup, remote homology detection is simulated by holding out all sequences of a particular SCOP family from a given superfamily in order to use these members as positive test examples. Positive training examples were selected from the remaining families in the same SCOP superfamily. Negative training and test examples have been drawn from disjoint sets of folds outside the fold of the target (test) family. In that way, every detection experiment involves a specific set of negative examples. According to the considered subset of SCOP families there are 54 binary classification problems at the superfamily level of the SCOP hierarchy. In this setup, the number of negative examples for each experiment is much larger than that of the positive ones. In particular, this situation gives rise to highly "unbalanced" training sets. In total, the setup consists of 4352 sequences from the SCOP 1.53 database.

To test the quality of our representation based on average word similarity, we utilize kernel-based support vector machines (SVM). Kernel methods in general require the evaluation of a kernel matrix including all inner products between training examples. To speed up computation, we pre-calculated the kernel matrices based on all 4352 sequences for different *K *and extracted the experiment-specific entries according to the setup of [6]. In the evaluation we tested our method for words of length *K *= 1, .., 10, whereby the entries of **K **= [*k*_*ij*_] were normalized according to

(15) $${k^{\prime }}_{ij}=\frac{k_{ij}}{\sqrt{k_{ii}\cdot k_{jj}}}.$$

All kernel matrices used for the evaluation can be downloaded in compressed text format from [17]. Instead of the GIST support vector machine that was used in the original setup, we apply a MATLAB^® ^implementation of the soft margin SVM with quadratic loss function as described in [18] for kernel-based training. The first reason is that we observed convergence problems of the GIST SVM in some cases. The second reason is that the direct implementation is considerably faster since the GIST package requires to create large experiment-specific data files containing the training and test kernel matrices. For reasons of comparability to the setup in [6], we used the same constant offset parameter (*o *= 10) for the kernel matrix and fixed the scaling parameter of the diagonal factor to a constant value (*q *= 1). While the offset parameter is added to all entries of the kernel matrix, the diagonal factor only affects the diagonal elements in order to cope with the unbalanced data sets [19]. With the diagonal factor *q *and the median of the diagonal elements *m*, $\frac{N^{+}}{N}\text{qm}$ and $\frac{N^{-}}{N}\text{qm}$ are added to diagonal elements for positive and negative examples, respectively. For training of the SVM we use the normalized kernel as defined in Equation (15) without any further transformations.

Besides from the unbalanced training sets, the setup in [6] also provides unbalanced test sets. In this case, widely-used performance metrics like predictive accuracy are not applicable [19]. Furthermore, homology search usually requires the analysis of an ordered list of potential homologs rather than hard classification. To measure the detection performance of our method on the test data, we calculated the area under curve with respect to the receiver operating characteristics (ROC) and the ROC50 score, which is the area under curve up to 50 false positives. Besides this, we also computed the median rate of false positives (mRFP). The mRFP is the ratio of false positive examples, which score equal or higher than the median score of true positives.

The results of our performance evaluation are summarized in Table 1 in comparison with other approaches. In order to exclude differences due to different implementation of the *L*_2_-SVM, we recalculated the detection performance for all approaches. For the Spectrum method, we also performed experiments with combined kernel matrices using word length sets $\hat{K}$ = {1, 2}, $\hat{K}$ = {1, 2, 3} and $\hat{K}$ = {1, 2, 3, 4}. For this purpose, we calculated the average kernel matrix element over different word lengths. The performance indices in the table correspond to average ROC/ROC50 and mRFP values over all 54 experiments. Furthermore, the average number of support vectors is given in the fifth column of the table. Support vectors are data examples with a non-zero weight after kernel-based training and have to be considered for kernel-based classification of new sequences. Therefore, a lower number of support vectors in general decreases the storage requirements and the computational demands for kernel-based evaluation of the discriminant. In addition, most SVM training schemes benefit from a smaller number of support vectors in terms of decreasing computation time.

**Table 1:** Overview of detection performance for several methods.

Method	avg. ROC	avg. ROC50	avg. mRFP	avg. # SV
*WCM*_1_	0.8705	0.3153	0.1065	1798
*WCM*_2_	0.8926	0.3814	0.0833	1673
*WCM*_3_	0.8964	0.4040	0.0813	1628
*WCM*_4_	0.9013	0.4257	0.0801	1604
*WCM*_5_	0.9032	0.4413	0.0795	1591
*WCM*_6_	0.9044	0.4473	0.0778	1591
*WCM*_7_	0.9036	0.4454	0.0785	1600
*WCM*_8_	0.9024	0.4470	0.0801	1607
*WCM*_9_	0.9018	0.4516	0.0815	1614
*WCM*_10_	0.9012	0.4528	0.0830	1620

LA-eig	0.9348	0.6614	0.0489	2640
ODH Monomer	0.9135	0.4554	0.0729	1601
SVM pairwise	0.9008	0.3986	0.0810	2355
Mismatch (5,1)	0.8852	0.3815	0.0949	2943
Spectrum (3)	0.8239	0.2939	0.1535	2350
Spectrum {1,2}	0.8919	0.3913	0.0798	1560
Spectrum {1,2,3}	0.8957	0.4094	0.0766	1711
Spectrum {1,2,3,4}	0.8981	0.4180	0.0769	1882

Performance evaluation results of the word correlation approach (*WCM*_*K*_) using several word lengths *K *= 1, ..10 in comparison to local alignment kernel (LA-eig) [10], Monomer Distance Histograms (ODH Monomer) [14], SVM pairwise [6], Mismatch string kernel [8], Spectrum kernel [9] and the combination of Spectrum kernels for different word lengths (see section "Results").

The performance values indicate that the WCM approach is well-comparable with other state-of-the-art methods. While the local alignment kernel and monomer distance histograms show better ROC and ROC50 performance, our new approach outperforms other feature-space based methods as well as the SVM pairwise kernel.

**Figure 2:**
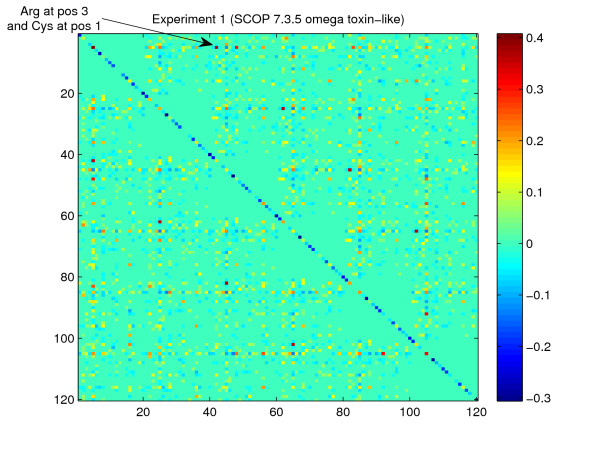
**Discriminant of SCOP superfamily 7.3.5 in the WCM space**. Word correlation matrix representation of the discriminant weight vector of superfamily 7.3.5 (omega toxin-like) after training using *K *= 6 (see text). Rows and columns correspond to occurrences of amino acids at two particular word positions for the first and second occurrence, respectively. Red (blue) matrix elements represent large positive (negative) discriminant weight values according to the color bar on the right hand side.

As described in the previous section, an explicit discriminant weight vector can be calculated in WCM feature space (see Equation (11)). Therefore, the weight vector can be visualized in WCM space for identification of discriminative features. Figure 2 shows the WCM discriminant of superfamily 7.3.5 (omega toxin-like) according to experiment 1 after kernel-based training using word length *K *= 6. Rows and columns correspond to particular amino acids at particular word positions for the first and second word occurrence, respectively. Elements with values in the range between 10% of the largest negative and 10% of the largest positive discriminant value were set to zero to reduce the noise in the visualization. Large positive values indicate that for detection of SCOP family 7.3.5.2 (Spider toxins) the corresponding feature is overrepresented in positive training sequences as compared with the negative training sequences. Table 2 shows a list of the 10 most discriminative words for the positive training sequences associated with superfamily 7.3.5 after kernel-based training (see section "Methods"). This table allows to identify the most discriminative features of a particular superfamily in biologically meaningful terms. For an exemplary analysis of globally important features, Table 3 shows the 10 most discriminative features of four experiments associated with families from the SCOP class "All alpha proteins". This class contains protein domains whose structure is essentially formed by alpha helices. The features in Table 3 correspond to particular dimensions in the word correlation feature space in terms of an amino acid pair at particular word positions.

**Table 2:** Ordered list of discriminative words for experiment 1.

#	Score	Word	Count
1	7.066	CCSGSC	3
2	6.930	CCSRKC	2
3	6.419	CRSGKC	4
4	5.451	CCRSCN	2
5	5.354	GRSGKC	1
6	5.215	CSRKCN	2
7	5.142	GRGSRC	1
8	4.979	CSGRGS	1
9	4.812	CCTGSC	4
10	4.789	SYNCCR	2

List of 10 most discriminative words for positive training sequences of experiment 1 according to SCOP superfamily 7.3.5 using word length *K *= 6. Words are sorted according to their word score. The first and second column correspond to rank and score of a word, respectively. The third column contains the word as amino acid sequence in IUPAC one-letter code. In the fourth column, the number of occurrences of a particular word in the positive training sequences are shown.

**Table 3:** Ordered list of discriminative features.

#	Family 1.27.1.1	Family 1.27.1.2	Family 1.36.1.2	Family 1.36.1.5
1	Leu@5, Leu@5	Leu@6, Leu@6	Thr@1, Val@5	Ala@1, Lys@5
2	Leu@6, Leu@6	Leu@5, Leu@5	Thr@2, Val@6	Ala@2, Lys@6
3	Leu@1, Leu@1	Leu@1, Leu@1	Val@1, Ser@2	asp@2, asp@2
4	Leu@2, Leu@2	Leu@2, Leu@2	Val@2, Ser@3	asp@3, asp@3
5	Leu@4, Leu@4	Leu@4, Leu@4	Val@5, Ser@6	asp@1, asp@1
6	Leu@3, Leu@3	Leu@3, Leu@3	Val@4, Ser@5	asp@4, asp@4
7	Leu@1, Leu@5	Leu@1, Leu@5	Val@3, Ser@4	asp@6, asp@6
8	Leu@2, Leu@6	Leu@2, Leu@6	Val@2, Thr@6	asp@5, asp@5
9	Glu@6, Glu@6	Glu@1, Glu@1	Val@1, Thr@5	Ala@1, Leu@2
10	gly@1, gly@1	Glu@2, Glu@2	Ser@1, Thr@4	Ala@2, Leu@3

List of 10 most discriminative features for four superfamilies associated with the SCOP class "All alpha proteins". Features are sorted in descending order according to their absolute discriminative weight (not shown). The first column corresponds to the rank of a feature and the succeeding columns contains the description of the feature in the word correlation feature space in terms of a pair of amino acids (in IUPAC three-letter code) at particular word positions. Features that are associated with negative discriminative weights are printed with lowercase first letters.

## Discussion

Table 1 indicates that the best ROC performance for the WCM approach on the SCOP benchmark setup is achieved using word length *K *= 6. For longer words, the ROC performance gradually decreases but still remains comparable with the other methods. However, the ROC50 performance for longer words increases and nearly achieves the ROC50 performance of the Oligomer Distance Histogram method for monomers. While prediction scores of all test examples are used for computation of the ROC performance, the ROC50 performance takes into account only prediction scores up to 50 false positive examples. This corresponds to an evaluation of the ROC curve in regions where a maximum number of 50 false positive examples are allowed for computation of specificity. Therefore, the results indicate that longer words yield more specific predictions. However, as compared with the local alignment kernel method [10] the WCM method performs inferior in terms of ROC and ROC50 scores. On the contrary, the detection performance of this approach depends on several hyperparameters. Table 1 shows that the performance of the WCM approach does not depend critically on the word length *K*. This obviates the tuning of this method parameter for different setups. However, longer words may be more suitable to identify biologically meaningful features or regions within sequences than short words.

### Comparison to closely related approaches

**Figure 3:**
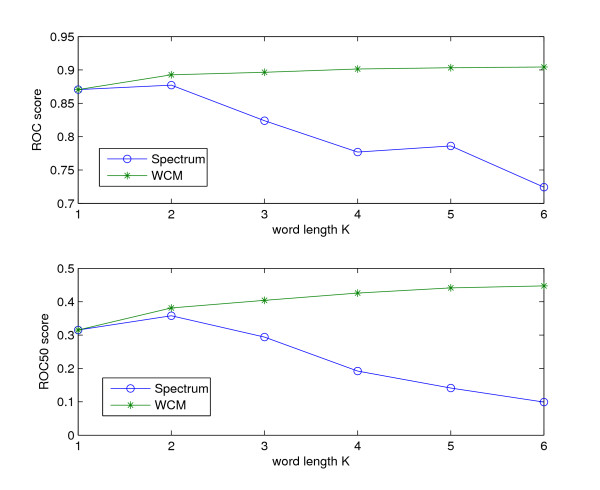
**Comparison of ROC and ROC50 performance for Spectrum method and WCM method**. The figure shows the mean ROC and ROC50 performance over 54 experiments for the Spectrum method and the word correlation method (WCM) using word length *K *= 1, .., 6.

Surprisingly, our WCM approach for *K *= 1 (*WCM*_1_) outperforms the *K*-mer Spectrum method for *K *= 3 (Spectrum (3)) in terms of ROC and ROC50 performance. Technically, the *WCM*_1 _feature space corresponds to the feature space of the Spectrum (1) method, i.e. the amino acid composition. This feature space comprises only 20 dimensions, and thus allows for fast and memory efficient representation and classification of sequences. This suggests that this simple approach could be useful for large-scale remote homology detection. In [9], the authors applied the Spectrum method to a similar remote homology detection setup as described here (see also [5]). However, the authors limit the evaluation of detection performance to the Spectrum (3) and Spectrum (4) method, respectively. Thereby, the Spectrum (3) method outperformed the Spectrum (4) method in terms of ROC50 performance. Figure 3 shows a comparison of the ROC performance for the Spectrum method and the WCM approach using word length *K *= 1, .., 6. It is clearly visible that the performance of the Spectrum rapidly decreases for growing word length while the performance of our method continuously increases. This results from the fact that the WCM feature space for a word length *K *> 1 completely includes the WCM feature space for shorter words. In contrast, the Spectrum feature space associated with a particular word length does not include the feature space for shorter words by definition. The results indicate that the Spectrum method is rather unsuitable for use of longer words. This can be traced back to the fact that the number of exact matches rapidly decreases for growing word length. This results in very small values for the similarity between two non-identical sequences. Therefore, the incorporation of inexact matches as in [8] is necessary for use with longer words. In [15], the authors present several string kernels that are based on inexact matching of *K*-mers. These methods realize inexact matching by a so-called "mismatch" or "mutation neighborhood" using a binary match function with specific invariance properties. In that case, a particular *K*-mer is mapped to several dimensions in the feature space of the *K*-mer Spectrum. The similarity of two *K*-mers can then be calculated as the dot product in this feature space. However, this feature space grows exponentially with *K *and is difficult to interpret in terms of biological sequence features. Furthermore, classification with the discriminant in this feature space for large *K *is demanding in terms of memory requirements. In contrast, the WCM method is based on a more "continuous" similarity measure between two words (see also equation (2)) rather than on a binary match criterion. The corresponding feature space only grows quadratically with *K *and each feature space dimension directly corresponds to a biologically meaningful sequence feature. In addition, the WCM approach allows for memory efficient classification with the discriminant in feature space.

Another possibility to deal with the decrease of exact matches for longer words is the combination of Spectrum kernel matrices based on different word lengths. Table 1 shows that the results for the Spectrum method using combined kernel matrices up to a maximum word length are only slightly inferior as compared with the WCM approach using the respective maximum word length. Note that the WCM approach does not require to identify a suitable combination of different kernels to achieve good prediction performance.

### Interpretation of discriminative features

The WCM feature space is useful for identification of discriminative features that have been learned from the data. In Figure 2, the discriminant weight vector is visualized in the WCM feature space that allows to analyze discriminative features in terms of the corresponding sequence properties. For example, the highlighted matrix element in Figure 2 indicates that for positive training sequences of superfamily 7.3.5 the occurrence of Cysteine (C) at the first word position in combination with Arginine (R) at the third word position is highly discriminative. This feature may not be detected in the sequences associated with this superfamily if only unsupervised methods, e.g. motif finders are used. The reason is, that the combination can only be observed in few cases but nevertheless occurs more often than in protein sequences from unrelated families. Therefore, our discriminative approach can help to identify features that are likely to be overlooked by unsupervised methods. These features can readily be used for analysis of more specific biological properties of the particular protein family.

Table 2 shows a list of the 10 most discriminative words in positive training sequences of superfamily 7.3.5 (omega toxin-like) after kernel-based training. Some of these words are very similar, e.g. words no. 1, 2, 4 and 9 begin with two Cysteine residues and words no. 1, 2 and 9 end with a Cysteine, too. Word no. 10 also shows two successive Cysteine residues, but in this case at word positions 4 and 5. The last column of Table 2 contains the number of occurrences of a particular word in the set of positive training sequences. It can be seen that this number is not directly related to the discriminative word score in the second column. This indicates that discriminative learning and unsupervised counting of words produce motifs with different meanings. The most discriminative word (CCSGSC) can easily be identified in the multiple alignment of the Omega-toxin family in the Pfam database [20]. The figure in Additional file 1 shows the full alignment of this family, which is a member of the omega toxin-like superfamily according to experiment 1 in the remote homology detection setup. In two sequences, the word exactly matches the subsequence and in 5 of the 6 remaining sequences the word only differs by one amino acid. In this case, exact word matches cannot capture the conserved region of the sequences. In contrast, the WCM method is able to capture this similarity in terms of high scoring words. Figure 1 shows score profiles of the first 5 positive test sequences associated with experiment 1 using word length *K *= 6. All score profiles have a global maximum that corresponds to a discriminative sequence region. For example, in sequence no. 5 the score maximum corresponds to the word CCSQSC, which is very similar to the most discriminative word in the training sequences. This indicates that score profiles may be used to identify characteristic sequence regions.

Table 1 shows that after kernel-based training the average number of support vectors of the WCM approach is significantly lower than that of the local alignment kernel and the Mismatch and Spectrum kernel methods. This may suggest that WCMs might be a more concise and accurate representation of globally important protein features such as secondary structure elements. Table 3 shows the most discriminative features of four protein families from the SCOP class "All alpha proteins". In the protein families 1.27.1.1 and 1.27.1.2 (long-chain/short-chain cytokines), the occurrences of Leucine at word position 1 and 5 (2 and 6) are among the top ten discriminative features. Similarly, in the protein families 1.36.1.2 and 1.36.1.5 (phage repressors/bacterial repressors) the occurrences of Valine at word position 1 and Threonine at word position 5 as well as the occurrences of Alanine at word position 1 and Lysine at word position 5 belong to the top ten discriminative features. This indicates that the characteristic distance of 4 residues between linked amino acids in an alpha helix provides a discriminative sequence feature in these families.

### Computational efficiency

In section "Methods", we pointed out that our WCM approach is very efficient in terms of computation time requirements for feature extraction from sequences. The feature-based calculation of the 4352 × 4352 kernel matrix for the WCM approach using word length *K *= 6 takes 31.62 seconds. This is by orders of magnitude faster than the computation of the kernel matrix for the local alignment kernel method, which nearly takes 2 hours. However, feature-based computation of the kernel matrix can also be applied to the Spectrum method. For *K *= 1 (*K *= 3), the calculation only requires 6.9 (10) seconds. For classification of new sequences with alignment-based kernel methods all kernel functions between the test sequences and support vector sequences, i.e. sequences with a non-zero weight after kernel-based training, have to be evaluated. For example, for classification of a new sequence with the local alignment kernel on average 2640 kernel function evaluations need to be computed. Using the software provided by the authors of [10], evaluation of a single kernel function requires on average 0.36 *ms *CPU time. In total, this yields 0.95 *s *for classification of a single sequence.

For classification of new sequences with the WCM approach, the discriminant weight vector in feature space can be used instead of the kernel-based evaluation. This dramatically reduces the computational effort for classification, because only transformation of the new sequence to a WCM feature vector and calculation of the dot product of that vector with the discriminant weight vector are necessary. If indicator vectors are used for amino acid representation, the score of a sequence can be computed by summing up all weight vector entries according to the number of occurrences of the associated pair of amino acids at two particular word positions in the sequence. We implemented a fast MATLAB^® ^version of this scoring procedure that requires on average 0.09 *ms *for scoring of a single sequence in the SCOP setup using word length *K *= 6. This is more than 10000 times faster than scoring with the local alignment kernel and implies a different category of computation time requirements for ranking of potential homologs in a large database. For example, the UniProt Protein Knowledgebase [21] release 12.8 contains 5678599 protein sequences, which could be potential targets in a homology detection task. In this case, scoring with the local alignment kernel would require more than 60 days on a single machine. Although not directly comparable in terms of detection performance, the feature-based scoring with the WCM approach takes less than 9 minutes. For comparison with the Spectrum method, we also implemented a fast procedure that scores a protein sequence using a feature space discriminant as produced by the Spectrum kernel method. For *K *= 1 (*K *= 3), scoring of the UniProt database takes about 4 (10) minutes. In principle, the computational cost for classification of new sequences with alignment-based kernels grows linearly with the number of training sequences. Therefore, the application of these methods to large-scale classification setups is problematic, too. In contrast, the computational cost for classification with the feature-based methods only grows linearly with the number of feature space dimensions. Therefore, our method is suitable for large-scale classification setups. In particular, the WCM approach could be very useful to reduce the number of target sequences or target families. This reduced set may then be further investigated with more specific alignment-based methods.

## Conclusion

In this work, we presented a new approach for protein sequence representation based on word correlation matrices (WCM). WCMs arise from a sequence kernel defined by average pairwise word similarity between two sequences. The approach shows comparable detection performance to state-of-the-art methods for protein remote homology detection. Our method includes a single kernel parameter that specifies the word length. We showed, that the detection performance does not critically depend on this parameter. Our results indicate, that for remote homology detection the word length parameter can be fixed to *K *= 6 for time and memory efficiency. Our protein sequence representation is associated with an explicit feature space in terms of word correlations. The discriminant weight vector in feature space can be used for fast classification of new sequences and intuitive interpretation of discriminative features.

In general, the basic word similarity measure can be defined in other ways than presented in this work. For example, in the definition of the word similarity measure (Equation (2) in section "Methods") a word substitution matrix can be inserted between the word vectors to include prior knowledge about the similarity of particular words. On the other hand, such substitution matrices are usually problem-specific, i.e. they should depend on the application. Furthermore, the substitution matrix has to be positive semidefinite so that the similarity measure still implies a valid sequence kernel.

Like other explicit feature space methods, our representation approach can be combined with different feature selection techniques. This would be useful in cases where a small set of relevant features has to be identified. Finally, the WCM approach is not limited to protein sequences, but can also be used for DNA or RNA sequence representation. In this case, the word length possibly has to be chosen larger to obtain meaningful features. The investigation of these possibilities will be part of future work.

## Authors' contributions

TL did the experimental evaluation and drafted parts of the manuscript. PM designed the method and drafted parts of the manuscript. Both authors read and approved the final manuscript.

## Supplementary Material

Additional file 1 **Pfam full alignment of the Omega-toxin family (PF06357)**. The file pfamAln.png contains a screenshot from the Pfam website (see [22]) which shows the multiple alignment of all member sequences of the Omega-toxin family (Pfam ID PF06357). The Omega-toxin family belongs to the omega toxin-like superfamily. Some of the discriminative words in Table 2 can be identified in the sequences (see text). Click here for file
