# Supplementary figures and images for: Word correlation matrices for protein sequence analysis and remote homology detection
Source: BMC Bioinformatics. 2008 Jun 3;9:259. doi: 10.1186/1471-2105-9-259 (PMC2438326; doi:10.1186/1471-2105-9-259)

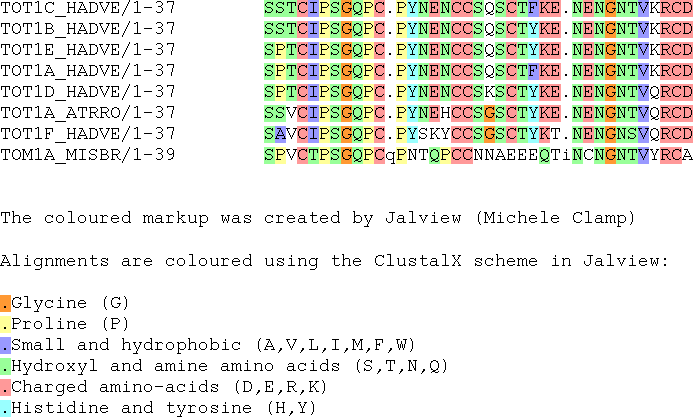

Supplement: Additional file 1 — Pfam full alignment of the Omega-toxin family (PF06357). The file pfamAln.png contains a screenshot from the Pfam website (see [22]) which shows the multiple alignment of all member sequences of the Omega-toxin family (Pfam ID PF06357). The Omega-toxin family belongs to the omega toxin-like superfamily. Some of the discriminative words in Table 2 can be identified in the sequences (see text). [file 1471-2105-9-259-S1.png]
